# Identification of a prognostic biomarker predicting biochemical recurrence and construction of a novel nomogram for prostate cancer

**DOI:** 10.3389/fonc.2023.1115718

**Published:** 2023-04-03

**Authors:** Zhaojun Yu, Haichao Chao, Fanghua Xu, Huanhuan Deng, Leihong Deng, Zhen Song, Tao Zeng

**Affiliations:** ^1^ Department of Urology, The Second Affiliated Hospital of Nanchang University, Nanchang, JiangXi, China; ^2^ Medical Department, Nanchang University, Nanchang, JiangXi, China; ^3^ Pathology Department, The People’s Hospital of Pingxiang, Pingxiang, JiangXi, China; ^4^ Department of Ultrasound, The First Affiliated Hospital of Nanchang University, Nanchang, JiangXi, China

**Keywords:** prostate cancer, biomarker, biochemical recurrence, nomogram, prognosis

## Abstract

**Background:**

Biochemical recurrence (BCR) is common in prostate cancer (PCa), but its prediction is based predominantly on clinicopathological characteristics with low accuracy. We intend to identify a potential prognostic biomarker related to the BCR and construct a nomogram for improving the risk stratification of PCa patients.

**Methods:**

The transcriptome and clinical data of PCa patients were obtained from TCGA and GEO databases. Differential expression analysis and weighted gene co-expression network analysis (WGCNA) were used to screen out differentially expressed genes (DEGs) related to the BCR of PCa. Cox regression analysis was further applied to screen out DEGs related to BCR-free survival (BFS). Time-dependent receiver operating curve (ROC) analysis and Kaplan–Meier (K-M) survival analysis were conducted to assess the prognostic value. Then, a prognostic nomogram was established and evaluated. The clinicopathological correlation analysis, GSEA analysis, and immune analysis were used to explore the biological and clinical significance of the biomarker. Finally, the qRT-PCR, western blotting, and immunohistochemistry (IHC) were conducted to validate the expression of the biomarker.

**Results:**

BIRC5 was identified to be the potential prognostic biomarker. The clinical correlation analysis and K-M survival analysis found that the BIRC5 mRNA expression was positively associated with disease progression and negatively associated with the BFS rate. Time-dependent ROC curves verified its accurate prediction performance. The GSEA and immune analysis suggested that the BIRC5 was related to immunity. A nomogram with an accurate prediction for BFS of PCa patients was constructed. qRT-PCR, western blotting, and IHC results validated the expression level of BIRC5 in PCa cells and tissues.

**Conclusion:**

Our study identified BIRC5 as a potential prognostic biomarker related to BCR of PCa and constructed an efficacy nomogram for predicting BFS to assist clinical decision-making.

## Introduction

Approximately 1.4 million new cases of prostate cancer (PCa) were diagnosed in 2020, making it the second most prevalent male cancer in the world; it’s also the fifth most common cause of cancer death in men worldwide, with approximately 375,000 new deaths in 2020. In more than half of the countries in the world, especially Northern America, Northern and Western Europe, and Australia, the most common malignancy in men is PCa (1). In 2022, the estimated number of new cases and death cases of PCa in the United States will be more than 260000 and 34000, respectively (2). Due to the popularity of PSA screening and the advancement of prostate needle biopsy technology, more and more patients with early-stage PCa are diagnosed. Although most of these patients can achieve good treatment efficacy after radical prostatectomy (3); however, about 20%-40% of clinically localized PCa still develop a biochemical recurrence (BCR) (4). The prognosis for these patients was often poor because they are more likely to develop distant metastases (5). In order to improve the prognosis of patients and reduce the disease burden of patients, it is essential to identify those patients who are at high risk of developing BCR.

However, current BCR risk prediction approaches primarily focus on clinicopathological characteristics, such as PSA, Gleason score, and pathological T stage (6, 7). The BCR risk stratification recommended by EAU guidelines included the Gleason score and prostate-specific antigen doubling time (PSA-DT); a clinical trial found that this risk stratification had moderate accuracy in predicting patient outcomes (8). Therefore, the existing BCR risk stratification strategy based on clinical parameters needs further improvement. Inappropriate risk stratification may lead to overtreatment, which increases the disease burden of patients and wastes medical resources. In the past decade, the rapid development of sequencing technologies and bioinformatics have made molecular testing based on next-generation sequencing (NGS) a vital component of diagnosis, prognosis, and treatment monitoring of disease (9, 10). In addition, the rapid development of AI technology plays an essential role in the development of medicine. Machine learning has increasingly apparent advantages in disease diagnosis and prognosis prediction, especially in medical image analysis (11–14). Machine learning combined with sequencing technology is rapidly driving the development of genomics.

Bioinformatics is being utilized more and more frequently in gene expression profiling to learn about the underlying biological mechanisms of illnesses and to identify biomarkers for disease, especially cancer (15, 16). Weighted gene co-expression network analysis (WGCNA) is often applied to discover tumor biomarkers because of its efficiency in building the associated networks and identifying hub genes (17, 18). In WGCNA, genes are clustered into a co-expression module according to expression patterns, and then the relationship between the module and clinical traits is quantified. Additionally, differential expression analysis is the most frequently used method to identify a biomarker, which is essential for understanding the potential mechanism of tumorigenesis. The above two approaches can be jointly utilized to further improve the identification capacity of highly related genes.

Currently, there is a lack of biomarkers that can effectively predict the BCR risk of PCa. Our research intends to identify a potential biomarker to classify PCa patients with different BCR risks for improving prognosis.

## Materials and methods

### Data collection and preprocessing

The gene expression profile and related clinical data of PCa were obtained from the TCGA database (https://portal.gdc.cancer.gov/) (accessed on 17 July 2022), the UCSC Xena database (https://xenabrowser.net/datapages) (accessed on 17 July 2022) and GEO database (https://www.ncbi.nlm.nih.gov/gds) (GSE46602) (accessed on 18 July 2022. The TCGA-PRAD dataset contained 52 normal and 501 PCa samples; the GSE46602 dataset included 14 normal and 36 PCa samples. All samples were from radical prostatectomy (RP). In the TCGA-PRAD dataset, we regarded the “days_to_first_biochemical_recurrence” as the time to BCR, and the “biochemical_recurrence” as BCR status. In the GSE46602, the clinical data had contained BCR status and BCR free time. The BCR-Free Survival (BFS) was defined as the time from RP to BCR. The inclusion criteria: Samples with complete and clear BCR information, include BCR status, time to BCR and follow-up time. The exclusion criteria: (a). Samples with fewer than 30 days of follow-up time. (b). Samples whose BCR status was unclear or missing. (c). Samples whose BCR free time was unclear or missing. The GPL570 (Affymetrix Human Genome U133 Plus 2.0 Array) was used to annotate the GSE46602 dataset. According to the corresponding annotation file, the probes were transformed into gene symbols, duplicate probes were removed, and the “Limma” package was applied to normalize the expression matrix.

### Differential expression analysis

In the TCGA-PRAD dataset, we performed the differential expression analysis between PCa tissues and adjacent tissues by the “DESeq2” package; in the GSE46602 dataset, we used the “Limma” package to perform the same analysis; the criteria of |log2 fold change (FC)|>1 and False Discovery Rate (FDR) <0.05 was utilized to screen out the differentially expressed genes (DEGs).

### Weighted gene correlation network analysis to identify BCR-related genes

The top 5000 genes from the TCGA-PRAD dataset and GSE46602 dataset were separately screened according to the median absolute deviation (MAD) value. On the basis of the expression data of the top 5000 genes, a scale-free gene co-expression network was constructed through WGCNA. First, based on the expression profile of the top 5000 genes, PCa patients with BCR were clustered hierarchically to remove outliers. Then, we applied the pickSoftThreshold function to select the best threshold for soft power to guarantee the establishment of scale-free networks based on Pearson’s correlation coefficient between genes. A hierarchical clustering analysis was used to classify genes into distinct modules, adjacency matrices with soft power thresholds were converted into a topological overlap matrix (TOM) and a 1-TOM dissimilarity matrix (deepSplit = 2, minModuleSize = 30). The genes that have yet to be allocated were placed in a grey module. Afterward, to assess the correlation between the module and clinical trait (BCR and BCR-Free), Module Membership (MM), the Pearson correlation coefficients between genes in module and module eigengene (ME), was calculated. Gene Significance (GS), the Pearson correlation coefficient of the gene and clinical trait, was also computed. The module with the highest correlation of clinical trait (BCR) was considered a candidate module, and the genes in the module were selected for further analysis.

### Cox regression analysis to identify the key gene

Firstly, we took the intersection of the DEGs and key module genes and drew a Venn diagram through the R package “Ven diagram.” Then, the Cox regression analysis was conducted on intersection genes in the TCGA-PRAD and GSE46602 datasets to screen out genes related to BFS of PCa patients. Finally, we crossed the screened genes’ intersection to get the final key gene.

### Evaluation and validation of the prognostic value of the key gene

Based on the median expression of the key gene in the TCGA-PRAD and GSE46602 datasets, we divided the patients into high and low-expression groups and compared the prognostic differences in BFS between the two groups by the Kaplan-Meier (K-M) method. In addition, we also analyzed the prediction accuracy of this key gene by time-dependent ROC curves and AUC values. For further validation of the prognostic value and predictive abilities of the key gene, we integrated the expression data of the key gene with the clinical data of the GSE70770 (GPL10558) dataset.

### Gene set enrichment analysis and immune analysis of the key gene

The comprehensive analysis was performed based on the TGGA database because it had the most comprehensive clinical data, and the amount of data was adequate. Firstly, we analyzed the differences in the mRNA expression of BIRC5 between different clinical subgroups by the R package “ggpubr.” Subsequently, the Gene Expression Profiling Interactive Analysis (GEPIA) was used to draw the K-M curves and respectively explored the relationship between the mRNA expression of BIRC5 and OS and the relationship between BIRC5 expression and DFS. GSEA is an effective method for understanding gene expression profiles and gaining insight into the biological mechanism. The R package “clusterProfiler” was used for GSEA to explore the KEGG pathways related to BIRC5 in the high- and low-expression group and screen the top five pathways, and the criteria p < 0.05, FDR < 0.25 and |NES|>1 was considered as statistical significance. Then, the differences in infiltrated immune cells between high and low expression groups were compared, and the correlation was analyzed between immune cells and BIRC5 expression. Pearson correlation analysis was performed to examine the correlation between the expression of BIRC5 and the expressions of immune checkpoint genes; p <0.001 was judged statistically significant. To further evaluate the reactivity of immunotherapy in high and low expression groups, we obtained the tumor immune dysfunction and exclusion (TIDE) score files of PCa from the TIDE website (http://tide.dfci.harvard.edu/) and showed the difference in TIDE score between the two groups by Violin plot.

### Construction and evaluation of Nomogram

To obtain a practical clinical tool that can be used to predict BFS in PCa patients, we integrated the mRNA expression data of BIRC5 and other clinicopathological factors to construct a nomogram. Since the TCGA-PRAD dataset has complete clinical information and a larger sample size than the GEO datasets, the nomogram construction was based on the TCGA-PRAD dataset. The univariate Cox regression analysis was applied to screen out prognostic factors correlated with the BFS of PCa (p<0.05); subsequently, the final prognostic factors were further screened by the least absolute shrinkage and selection operator (LASSO) regression analysis. Based on the above factors, a nomogram was developed to predict BFS in PCa patients. C-index analysis, ROC analysis, DCA analysis and K-M survival analysis were conducted to evaluate the predictive performance and stability of the nomogram. The above analysis processes were completed by R package “survival”, “survminer”, “rms”, “ggDCA”, “timeROC”.

### Cell culture

The immortalized prostate luminal epithelial cell line RWPE1, and PCa cell lines PC3 and DU145, were acquired from ATCC. PC3 and DU145 cells were grown in Ham’s F-12K medium (Procell) and RPMI1640 medium (Invitrogen), respectively. RWPE1 cells were cultured in Defined Keratinocyte‐SFM medium (ThermoFisher). All medium was supplemented by 10% FBS and 1% P/S (penicillin/streptomycin); all cells were grown in 37°C with 5% CO2.

### qRT-PCR

TRIzol reagent (Invitrogen, USA) was used to extract total RNA from RWPE1, PC3, and DU145 cells. We used ACTIN as an internal control. In order to calculate the relative expression, we used the 2−△△Ct method. As shown in **Supplementary Table S1**, the primer sequences are listed.

### Western blotting

In order to analyze the protein expression level of BIRC5 in PCa cells, we performed western blotting analysis. Total protein was extracted from cells using RIPA buffer, and protein concentration was determined using the BCA method. The protein samples were boiled in an SDS-PAGE loading buffer before being separated by 12% SDS-PAGE, and a PVDF membrane was then used to transfer the protein. After using 5% skim milk to block PVDF membranes for 1.5h, Membranes were incubated with corresponding primary antibodies (BIRC5: 1:1000, Gapdh: 1:1000) and secondary antibodies, respectively. Finally, the protein bands were visualized by chemiluminescence kits and analyzed by Image J.

### Tissue samples and IHC analysis

Ten para-cancerous normal tissue samples and thirty PCa tissue samples were collected from tissue biobank in the Second Affiliated Hospital of Nanchang University. In addition, twelve paired PCa tissues and the adjacent normal tissues were also obtained from PCa patients who received prostate needle biopsies. The Ethics Committee approved the study prior to the start of the research. For immunohistochemistry staining, 4-um thick sections were cut from paraffin-embedded specimens. For antigen retrieval, sections were deparaffinized with xylenes, rehydrated, and incubated in EDTA at 120°C for 10 minutes. 3% H2O2 in distilled water was applied to the sections for 10 minutes in order to quench the endogenous peroxidase activity, followed by an incubation with 5% goat serum in TBS for 1.5 hours to prevent nonspecific binding. A primary antibody was then incubated overnight at 4°C with tissue sections (Survivin 1:300, 10508-1-AP, Proteintech, China). After rinsing the slides three times with TBS, the slides were incubated with a peroxidase-labelled secondary antibody (Proteintech, China). 3-diaminobenzidine tetrahydrochloride (DAB) was utilized to visualize the immune complexes, followed by hematoxylin counterstaining. The IHC slides were scanned by The Aperio AT2 scanner (Leica), and the Image J software was used to measure the average integrated optical density (AOD) of the IHC image. AOD = integrated optical density (IOD)/Area. We assessed IHC staining intensity by calculating AOD values.

### Statistics

**Figure 1:**
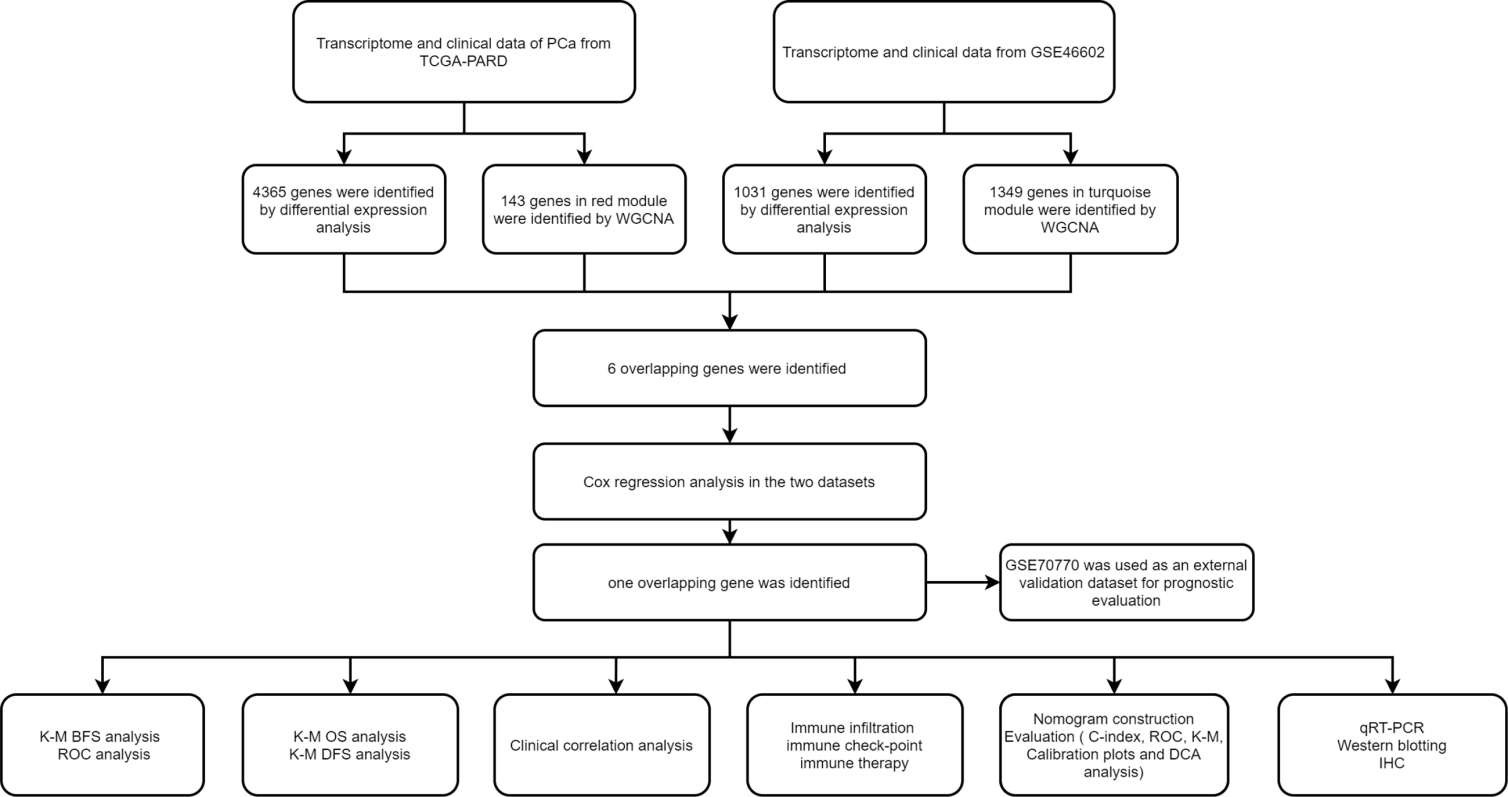
The flowchart of our study.

The above-mentioned R packages (R version 4.1.3.) and GraphPad Prism 7 were used to perform statistical analyses. Student t-tests and Wilcoxon tests were utilized to analyze continuous variables. In the Cox regression analysis and the nomogram analysis, categorical variables were calculated and classified into 0, 1, 2, 3, and 4. The *p* < 0.05 was defined as the statistical significance. As shown in **Figure 1**, we outlined the process of our study.

## Results

### Identification of the differentially expressed genes

**Figure 2:**
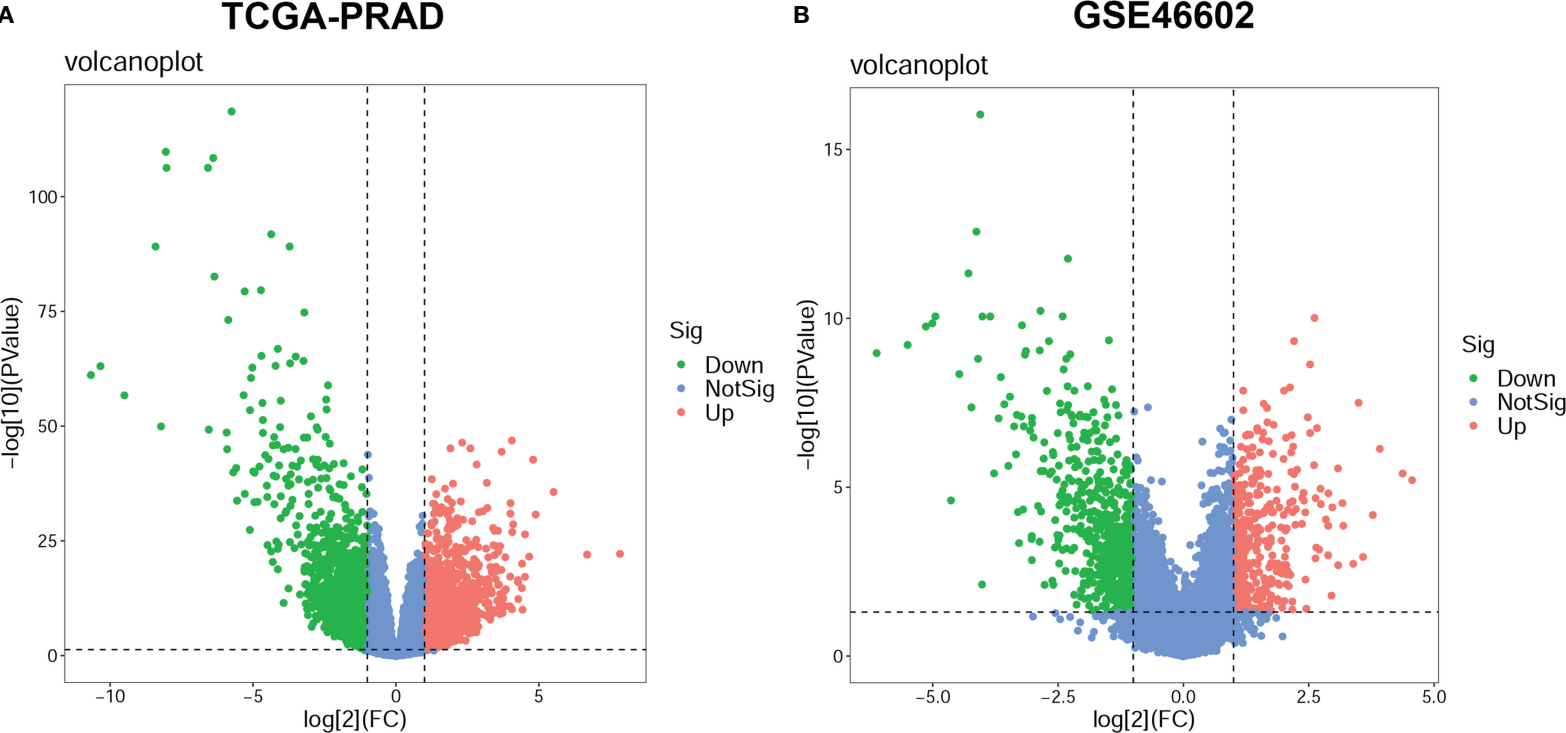
Differentially expressed genes (DEGs) between prostate cancer (PCa) and normal prostate tissue. The volcano plots for DEGs in the TCGA-PRAD dataset **(A)** and the GSE46602 dataset **(B)**. The significant up-regulated genes are indicated by red dots; The significant down-regulated genes are indicated by green dots; and the nonsignificant genes are indicated by blue dots.

In the TCGA-PRAD dataset, differential expression analysis identified 4365 DEGs between normal prostate tissues and PCa tissues, including 2165 up-regulated genes and 2200 down-regulated genes; while in the GSE46602 dataset, 1031 DEGs were identified, including 382 up-regulated genes, 649 down-regulated genes (**Supplementary Table S2**). The DEGs in the two datasets were shown in the volcano plot (**Figures 2A, B**).

### WGCNA to identify BCR-related genes

**Figure 3:**
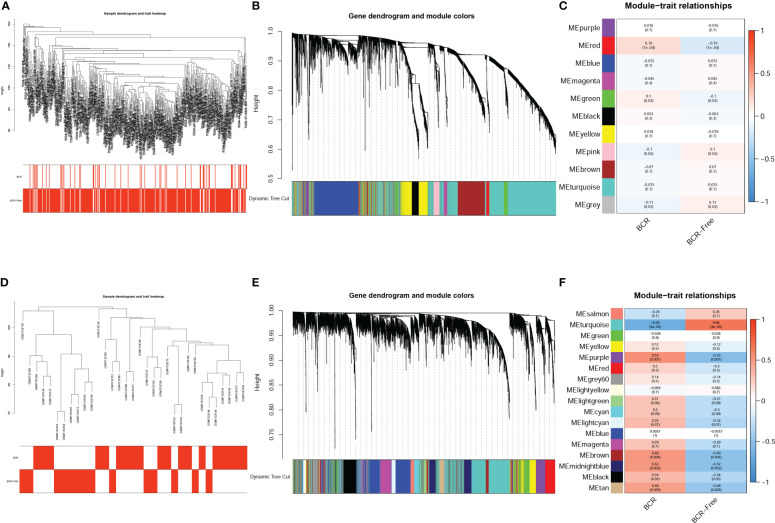
Identification of co-expressed modules related to biochemical recurrence (BCR). A cluster tree of PCa samples with a color band displaying clinicopathologic values beneath the tree in the TCGA-PRAD dataset **(A)** and GSE46602 dataset **(D)**. The cluster dendrogram indicated the different gene modules in the TCGA-PRAD dataset **(B)** and GSE46602 dataset **(E)**. The correlation coefficients between the modules and clinical features in the TCGA-PRAD dataset **(C)** and GSE46602 dataset **(F)**.

**Figure 4:**
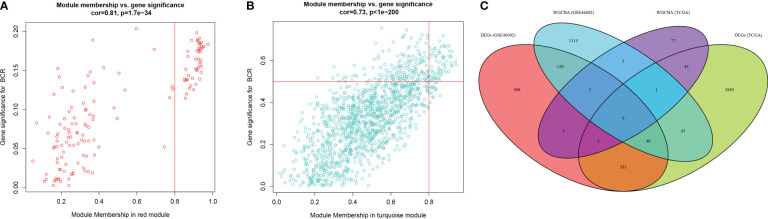
Differentially expressed genes (DEGs) correlated with biochemical recurrence (BCR). **(A, B)** Gene correlation scatter plots of the red module in the TCGA-PRAD dataset **(A)** and the turquoise module of the GSE46602 dataset **(B)**. Module membership is represented by the X-axis, and the significance of the gene is represented by the Y-axis. **(C)** Venn diagrams of DEGs and co-expressed genes of TCGA-PRAD dataset and GSE46602 dataset.

The expression matrix of top 5000 genes was turned into a gene co-expression network to explore the relationship between top 5000 genes (**Supplementary Table S3**) and the BCR status of PCa patients in TCGA-PRAD (**Figure 3A**) and GSE46602 datasets (**Figure 3D**). Cluster analysis was performed before WGCNA to ensure no outlier samples. It was found that there were three samples in each dataset with a distant clustering relationship in the two datasets. They were identified as outliers in the two datasets, respectively, and they were removed from our analysis (**Supplementary Figure S1A, B**). For network construction, we selected three as the optimal soft threshold for the TCGA-PRAD dataset and five as the optimal soft threshold for GSE46602 (**Supplementary Figures 1C, D**). Eleven co-expressed modules were identified in TCGA-PRAD dataset through a dynamic pruning method (**Figure 3B**). Seventeen co-expressed modules were identified in the GSE46602 dataset using the same method (**Figure 3E**). Subsequently, we analyzed the relationship between non-grey modules and clinical feature BCR. It was found that the red module in the TCGA-PRAD dataset and the turquoise module in GSE46602 had the highest correlation coefficients (**Figures 3C, F**). In the TCGA-PRAD dataset, the highest correlation between Module membership and gene significance existed in the red module with 143 genes (**Figure 4A** and **Supplementary Figure S2A-S2B**); in GSE46602 dataset, the highest correlation existed in the turquoise module with 1349 genes (**Figure 4B** and **Supplementary Figure S2C-S2F**). The genes from the two modules were selected for further analysis (**Supplementary Table S4**).

### Identification of the key gene-BIRC5

**Figure 5:**
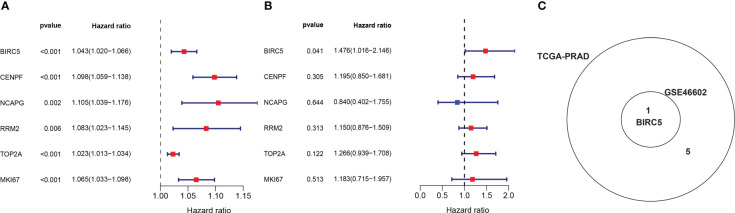
Identification of the final key gene. **(A, B)** The univariate Cox regression analysis in the TCGA-PRAD dataset **(A)** and the GSE46602 dataset **(B)**. **(C)** Venn diagram of prognostic-related genes from the GSE46602 dataset and the TCGA-PRAD dataset.

In order to obtain the DEGs related to the BFS of PCa, we intersected DEGs from the TCGA and GSE46602 datasets with genes from the TCGA dataset’s red module and GSE4660 dataset’s turquoise module. It was found that there were six genes in the intersection (**Figure 4C**). These genes were then subjected to a univariate Cox regression analysis in order to screen out DEGs related to BFS. The results showed that all six genes were correlated to BFS in the TCGA-PRAD dataset and only one gene in the GSE46602 dataset (**Figures 5A, B**). BIRC5 was identified as the final key gene by intersecting the genes related to BFS from the two datasets (**Figure 5C**).

### Evaluation and validation of the prognostic value of BIRC5

**Figure 6:**
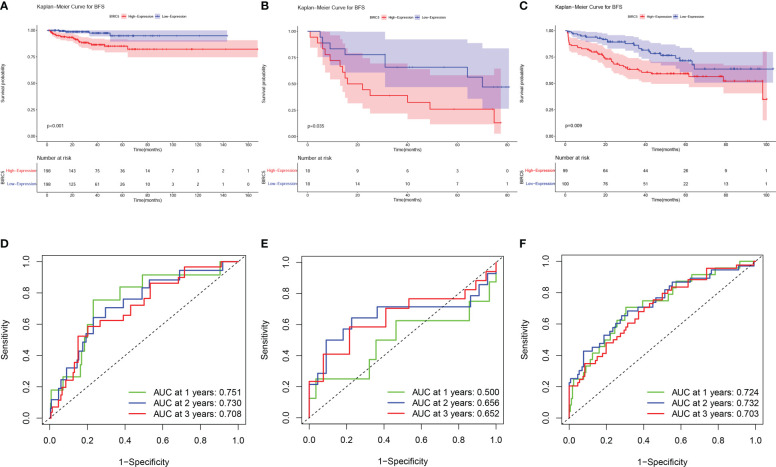
Prognostic evaluation of BIRC5 expression in various datasets. The K-M survival analysis indicated that patients with low expression of BIRC5 had a better BFS than those with high expression of BIRC5 in TCGA-PRAD dataset **(A)**, GSE46602 dataset **(B)** and GSE70770 dataset **(C)**. Time-dependent ROC curves demonstrated that the mRNA expression of BIRC5 acted as a powerful predictor of BFS for prostate cancer (PCa) patients in the TCGA-PRAD dataset **(D)**, GSE46602 dataset **(E)** and GSE70770 dataset **(F)**.

The BCR data of patients selected in the three datasets was shown in **Supplementary Table S5**. Based on the median expression of BIRC5, patients in all sets were classified into high-expression and low-expression groups. K-M curves were applied to evaluate differences in BFS between high- and low-expression groups. The results showed that patients with high BIRC5 expression had significantly lower BFS than patients with low BIRC5 expression in both the TCGA-PRAD dataset and the GSE46602 dataset (**Figures 6A, B**). The finding of K-M analysis in the GSE70770 dataset further verified that patients with high BIRC5 expression had poor BFS compared to low BIRC5 expression (**Figure 6C**). The time-dependent ROC curves showed that AUC values in the three datasets were all higher than 0.65, except for the AUC value of 1-year in the GSE46602 dataset (**Figures 6D–F**). The results of the ROC analysis proved the accurate prediction ability of BIRC5. By using the GEPIA database, we also evaluated the correlation between BIRC5 expression and disease-free survival (DFS) or overall survival (OS). A high level of BIRC5 expression was associated with a worse OS or DFS (**Supplementary Figure S3**).

### Clinical correlation analysis

**Figure 7:**
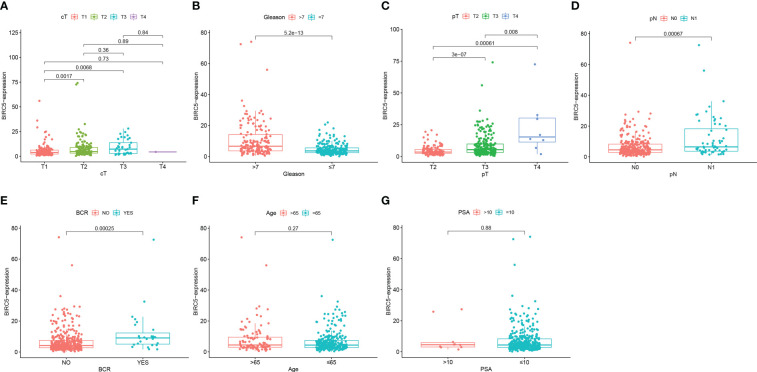
The correlation analysis between the mRNA expression of BIRC5 and different clinical characteristics. **(A)** Clinical T-stage. **(B)** Gleason score. **(C)** pathological T-stage. **(D)** pathological N-stage. **(E)** biochemical recurrence (BCR) status. **(F)** Age. **(G)** PSA.

We examined the correlation between BIRC5 expression and clinical features for better understanding the clinical relevance of BIRC5 in PCa. The detailed information about the selected patients was shown in **Table 1**. We found that patients with advanced clinical T-stage, pathological N-stage, pathological T-stage and Gleason score displayed higher BIRC5 expression (**Figures 7A–D**). In addition, the patients who developed BCR had higher BIRC5 expression than those who didn’t developed BCR (**Figure 7E**). However, we found no clear relation between the expression of BIRC5 and PSA and age (**Figures 7F, G**).

**Table 1:** Clinicopathological characteristics of patients selected in TCGA-PRAD dataset.

Clinicopathological characteristics	Patients		Clinicopathological characteristics	Patients		
Age (year)	60.85 ± 6.77		PSA			
Clinical T-stage			≤10	374	(94.44%)	
T1	146	(36.87%)	>10	10	(2.53%)	
T2	142	(35.86%)	NA	12	(3.03%)	
T3	37	(9.34%)	Radiotherapy	50	(12.63%)	
T4	1	(0.25%)	YES	284	(71.72%)	
NA	70	(17.68%)	NO	62	(15.65%)	
Gleason score			NA			
6	39	(9.85%)	Drugs information			
7	195	(49.24%)	Hormone Therapy	66	(16.67%)	
8	51	(12.88%)	Chemotherapy	1	(0.25%)	
9	108	(27.27%)	NA	329	(83.08%)	
10	3	(0.76%)	Pathological T-stage			
Pathological N-stage			T2	149	(37.63%)	
N0	284	(71.72%)	T3	234	(59.09%)	
N1	60	(15.15%)	T4	8	(2.02%)	
NA	52	(13.13%)	NA	5	(1.26%)	

NA indicates a null value.

### GSEA and immune analysis

**Figure 8:**
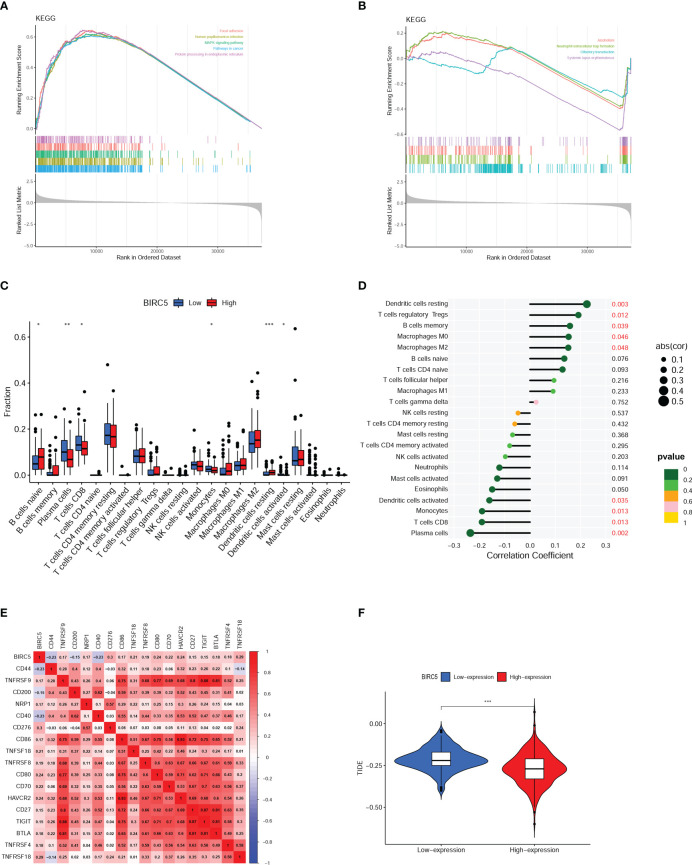
Gene set enrichment analysis (GSEA) and immune analysis. **(A, B)** GSEA pathway enrichment analysis of BIRC5 in prostate cancer (PCa) patients. **(C)** Immune cell infiltration in high- and low-expression of BIRC5 groups. **(D)** Correlation analysis between the mRNA expression of BIRC5 and immune cells infiltrated. **(E)** The correlation analysis between the BIRC5 expression and immune checkpoints. **(F)** The differential analysis of Tumor Immune Dysfunction and Exclusion (TIDE) scores between the low- and high-BIRC5 expression of PCa. (^∗^p < 0.05, ^∗∗^p < 0.01, and ^∗∗∗^p < 0.001).

As shown in **Figure 8A**, the main pathways correlated with high expression of BIRC5 were focal adhesion, human papillomavirus infection, protein processing in endoplasmic reticulum, pathways in cancer, and MAPK signaling pathway and the main pathways related to low expression of BIRC5 were alcoholism, neutrophil extracellular trap formation, olfactory transduction, systemic lupus erythematosus (**Figure 8B**). These pathways are primarily involved in Signal transduction, Genetic Information Processing, Immune, and Sensory systems.

Boxplots were used to compare the differences in the 22 different kinds of immune cells infiltrated. It was found that there was a distinct difference between the high and low BIRC5 expression in the immune cell subtypes, including plasma cells, naïve B cells, CD8 T cells, resting dendritic cells, activated dendritic cells and monocytes (**Figure 8C**). The result of correlation analysis demonstrated that the BIRC5 expression was positively related with resting dendritic cells, macrophages M0, memory B cells, T cells regulatory Tregs and macrophages M2; and BIRC5 expression was negatively related with activated dendritic cells, monocytes, CD8 T cells and plasma cells (**Figure 8D**). In the immune checkpoint correlation analysis, we found that BIRC5 expression most strongly correlated with CD276 expression, followed by TNFRSF18, CD80, and HAVCR2 (**Figure 8E**). TIDE analysis predicted that patients with high expression of BIRC5 would respond much more favorably to immunotherapy than those with low expression (**Figure 8F**).

### Nomogram construction and evaluation

**Figure 9:**
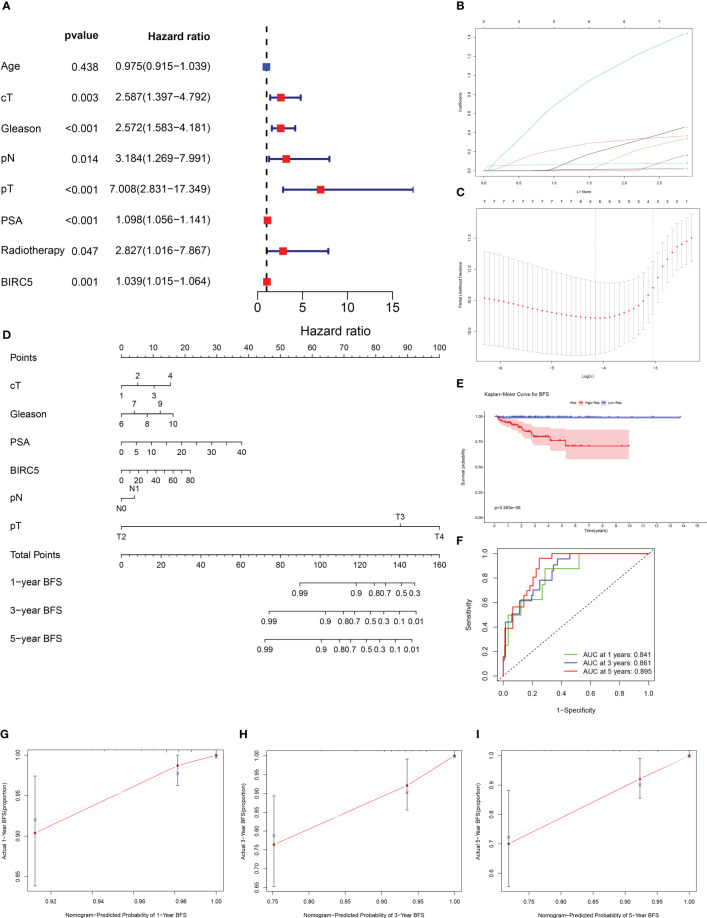
Establishment of a nomogram to predict biochemical recurrence-free survival (BFS) in PCa patients. **(A)** The univariate Cox regression analysis of clinicopathological characteristics and the mRNA expression of BIRC5. **(B, C)** The LASSO regression analysis identified the final prognostic factors. **(D)** A prognostic nomogram predicting 1-, 3-, and 5-years BFS of PCa patients. cT (clinical T-stage):1-T1, 2-T2, 3-T3, 4-T4. **(E)** The K-M survival analysis showed that patients with high risk had significantly shorter BFS than those with low risk. **(F)** The time-dependent ROC curves demonstrated the accurate prediction performance of the nomogram. **(G–I)** The calibration plots for 1-, 3-, and 5-year BFS of PCa patients.

By incorporating multiple risk factors, a nomogram is an invaluable tool for quantifying personal risk in clinical practice. Based on univariate Cox regression analysis, prognostic factors associated with BCR were screened out in PCa patients, including clinical T-stages, pathological N-stages, Gleason scores, pathological T-stages, PSA levels, radiotherapy, and BIRC5 mRNA expression (**Figure 9A**). The LASSO Cox regression analysis was applied to select variables to establish a nomogram. The result of LASSO regression analysis showed that when Log(λ) was about -4.20, the partial likelihood deviation was the most minor, and six variables were included (**Figures 9B, C**). Subsequently, we constructed a nomogram that integrated the six variables, including clinical T-stage, pathological T-stage, PSA, pathological N-stage, Gleason score, and the mRNA expression of BIRC5, to predict the BFS in PCa patients (**Figure 9D**).

Based on the median of nomogram computed-risk score, patients were categorized into high- and low-risk groups. In K-M survival analysis, patients with high-risk had significantly poorer prognosis than those with low-risk (**Figure 9E**). The nomograms’ predictive power was evaluated by drawing time-dependent ROC curves and computing AUC values. The result illustrated that the AUC values for 1-, 3-and 5-year BFS rate were 0.841, 0.861 and 0.895, respectively (**Figure 9F**). The calibration plots for the 1-, 3-, and 5-year BFS predictions demonstrated that the model’s predictions were in high agreement with the ideal outcomes (**Figures 9G–I**). The C-index analysis showed that the C-index value was 0.831 (95% CI: 0.788-0.874). Moreover, the 1-, 3- and 5-year DCA plots demonstrated that patients who use the model to forecast their prognosis might gain a good net benefit, indicating that the nomogram model had robust, practical application (**Supplementary Figure S4**).

### Experiment validation

**Figure 10:**
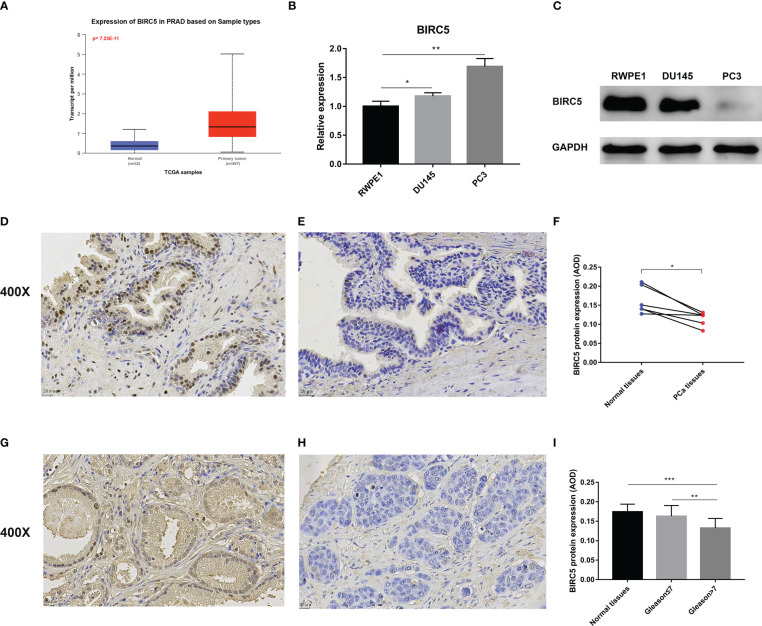
Validation of the mRNA and protein expression of BIRC5 in prostate cancer (PCa). **(A)** The mRNA expression of BIRC5 in PCa tissues was significantly higher than in normal tissues in the UALCAN database. **(B)** The mRNA expression of BIRC5 in PCa cells (DU145 and PC3) was significantly increased than in the prostate normal epithelium cells (RWPE1). **(C)** The protein expression of BIRC5 in PCa cells (DU145 and PC3) was significantly lower than that in RWPE1 cells. **(D, E)** The two Immunohistochemistry (IHC) images from the same patient. The normal prostate tissue (400X, bar = 20 um) **(D)**, the PCa tissue (400X, bar =20 um) **(E)**. **(F)** IHC analysis demonstrated that the BIRC5 staining in the normal prostate tissues was stronger than the paired PCa tissues. **(G)** The PCa tissue with a Gleason score 6 (400X, bar = 20 um). **(H)** The PCa tissue with Gleason score 9 (400X, bar = 20 um). **(I)** The staining of BIRC5 in PCa tissues with a Gleason score ≤7 was stronger than PCa tissues with a Gleason score >7 (^∗^P < 0.05, ^∗∗^P < 0.01, and ^∗∗∗^P < 0.001).

We investigated the BIRC5 mRNA expression through the UALCAN database and found that PCa tissue expresses significantly higher levels of BIRC5 mRNA than normal tissue (**Figure 10A**). Then, the BIRC5 mRNA expression in cells was explored by qRT-PCR. In PCa cells DU145 and PC3, BIRC5 mRNA expression was higher than in normal prostate epithelium cells RWPE1 (**Figure 10B**). In addition, we conducted a western blot assay to detect the protein expression of BIRC5. We found that RWPE1 cells expressed higher levels of BIRC5 protein than DU145 or PC3 cells (**Figure 10C**). IHC analysis showed that the staining intensity of BIRC5 in normal tissues was higher than that in paired PCa tissues (**Figures 10D–F**). In addition, we found that the staining intensity of BIRC5 was higher in tissues with Gleason score ≤7 than in those with Gleason score>7 (**Figures 10G–I**).

## Discussion

PCa is one of the most frequent malignancies in the world, and the high rate of BCR is its distinguishing feature. Approximately 34%, 44%, and 52% of patients with localized PCa will develop BCR at 10, 15, and 20 years after radical prostatectomy (19). BCR is an indication of clinical progression, once patients develop BCR, the risk of tumor metastasis will be significantly increased, and the prognosis of metastatic PCa patients is poor (20). Therefore, early identification of patients who are at high risk for BCR is vital to improving their prognosis. However, BCR predictions are primarily based on clinical features, which are not entirely accurate. With the rapid development of genomics and bioinformatics, the single use of clinicopathological parameters to predict the clinical outcome of patients is weak. It is becoming increasingly important to use genomics and clinicopathology together to identify biomarkers and predict clinical outcomes. To be able to predict the outcome of PCa patients, it is necessary to identify an effective prognostic biomarker.

From the TCGA and GEO databases, we collected gene expression profile and corresponding clinical data of patients with PCa, and six DEGs associated with PCa BCR were identified. After that, the correlation between these six genes and BFS was examined using Cox regression analysis and finally found the key gene BIRC5 with prognostic significance in both GSE46602 and TCGA-PRAD datasets. Notably, in Cox regression analysis, all of these six genes were associated with prognosis in TCGA-PRAD dataset, but only one gene was associated with prognosis in GSE46602 dataset. The reason for this difference may be due to the large difference in sample size between the two datasets and the large difference in the proportion of samples with BCR in the total samples. From another perspective, the differences in sample size and sample composition further indicate that the two datasets are completely independent, and BIRC5 was found to be associated with BFS in the two completely independent datasets, which further implies that BIRC5 is strongly associated with the prognosis of PCa patients.

The GSE70770 dataset was utilized as an external validation set, and the prognostic significance of BIRC5 was assessed and validated by the K-M method and time-dependent ROC curves on the three independent datasets. K-M curves suggested that the higher the level of BIRC5 mRNA expression, the more likely patients were to have a poor prognosis; meanwhile, BIRC5 was found to have good accuracy in predicting BFS through time-dependent ROC curves and corresponding AUC values. In the GSE46602 dataset, time-dependent ROC curves revealed that the 1-year AUC value for patients was 0.500, whereas the counterpart AUC values in the other two datasets exceeded 0.7. This difference may be due to the small amount of sample data in the GSE46602 dataset, with only 36 tumor samples, and the large amount of sample data in the other two datasets, among which GSE70770 dataset contains 199 tumor samples and TCGA-PRAD dataset contains 396 samples. Based on the results of K-M survival analysis and ROC curve analysis in the three datasets, BIRC5 was a biomarker that could effectively predict the BFS of PCa. Since the TCGA-PRAD dataset has more complete data and a larger number of samples than the GEO datasets, we subsequently performed a comprehensive analysis of BIRC5 through the TCGA-PRAD dataset to assess the biological and clinical significance of BIRC5.

Through the UALCAN database, BIRC5 mRNA was found to be highly expressed in PCa tissues, qRT-PCR results of cell lines also validated that BIRC5 mRNA expression was elevated in PCa cells. However, BIRC5 had low protein expression levels in PCa cells and tissues. Interestingly, the IHC result suggests that the BIRC5 protein expression in PCa tissues reduced with the increase of the Gleason score. Subsequently, through clinical correlation analysis of BIRC5, we found that in pathological N-stage, clinical T-stage, pathological T-stage, and Gleason score, the BIRC5 mRNA expression increased with the higher stage or Gleason score. Koike et al. reported that the BIRC5 mRNA expression was positively correlated with the Gleason score in PCa tissue samples (21), which validated our findings. The role of BIRC5 in tumor growth and development has also been identified in other cancers, including lung, brain and colon (22–25).

Then we conducted a GSEA analysis and found that BIRC5 was strongly correlated with Immune system-related pathways. Further immune analysis discovered that the BIRC5 mRNA expression was associated with the presence of immune cells in the tumor and the expression of immune checkpoint genes, indicating that BIRC5 may influence the immunological condition of PCa patients. In immune infiltration analysis, the results indicated that patients with high BIRC5 expression had lower CD8 T cell infiltration in tumor samples. Multiple studies have shown that as the main effector immune cells, CD8 T cells were essential for carcinogenesis and progression and played a crucial role in antitumor actions (26–28). Yanai et al. reported that PCa patients with a higher percentage of CD8 T cells in the immune microenvironment had a longer BCR (29). In this study, the infiltration degree of CD8 T cells was lower in the BIRC5 high-expression group, and the BFS was poorer in the BIRC5 high-expression group. The results were in line with previous research findings, which further suggest that BIRC5 plays a significant role in the development of PCa and may affect the immune microenvironment.

To obtain a practical clinical tool for predicting BFS of patients with PCa to help clinicians make a clinical decision. We successfully constructed an effective nomogram model by integrating BIRC5 mRNA expression and other clinical indicators related to PCa progression. Based on the K-M curve analysis, patients with high nomogram scores were more likely to have a worse BFS rate, meanwhile calibration plots showed a good prediction performance of the nomogram model. In addition, the time-dependent ROC curve of patients illustrated that the AUC values for 1-, 3-, and 5-year were all higher than 0.8, demonstrating this nomogram model’s high predictive efficiency.

BIRC5, the gene that encodes Survivin, is a member of the inhibitor of apoptosis (IAP) gene family, which plays a vital role in preventing cell death through apoptosis (30). Clinical correlation analysis showed that BIRC5 mRNA expression was related to the stage and grade of PCa. At the same time, the immune analysis showed that BIRC5 was related to the tumor microenvironment, especially CD8 T cell infiltration. Ma et al. found that BIRC5 is highly expressed in lung cancer, and it can promote the proliferation of tumor cells by regulating the expression of PD-L1 and tumor immune microenvironment. Zhao et al. also found that BIRC5 could promote the development of tumor cells by regulating the inflammatory tumor microenvironment in penile cancer (31). Although it has been confirmed that BIRC5 can affect the occurrence and development of tumors by affecting the tumor microenvironment in a variety of tumors, it is still a lack of literature to report whether BIRC5 also affects the development of PCa by affecting the tumor microenvironment. Our research suggested that BIRC5 might also contribute to PCa progression by altering the immune microenvironment of tumor cells. In addition, this study identified BIRC5 as a biomarker for predicting BCR in PCa patients through a series of bioinformatics methods. We verified its accurate prediction ability in three independent datasets from different databases. BIRC5 may be used as a potential biomarker for risk stratification of PCa patients to identify the population with high BCR risk early to improve patients’ prognosis. The nomogram based on BIRC5 mRNA expression is also expected to be a practical clinical tool for assessing BCR risk in PCa patients due to its good prediction efficiency and help urologists in clinical decision-making.

Although this study identified a prognostic biomarker associated with BCR of PCa through multiple analytic methods, there are still limitations. Firstly, although the prognostic value of BIRC5 was evaluated and validated in three independent datasets, the results are based on retrospective data. More prospective data are necessary to demonstrate the prognostic value of the biomarker and the nomogram. Secondly, the GSE46602 dataset is from Denmark, the GSE70770 dataset is from the United Kingdom, and the TCGA-PRAD dataset is from America. These three independent datasets used in our study are all from Western countries. Due to the differences in lifestyle habits and PCa epidemiology between Asian and Western countries, it is necessary to collect further data to verify whether our findings also apply to Asian countries. Thirdly, although BIRC5 does affect the development of tumor by affecting the tumor microenvironment in lung and penile cancer, our findings also predicted that BIRC5 may affect immune infiltration in PCa. However, since this result is based on bioinformatics, whether BIRC5 is also involved in PCa progression by affecting the tumor microenvironment still needs to be verified experimentally. In addition, whether the immune microenvironment affects the expression of BIRC5 also requires further experimental exploration.

## Conclusion

Through the differential expression analysis, WGCNA analysis, and Cox regression analysis, our research identified a significant prognosis-related biomarker, BIRC5, which accurately predicted BFS in PCa patients and a high correlation with immune infiltrations. A nomogram integrated BIRC5 mRNA expression and other clinicopathological features was also successfully constructed and could be used as a practical tool for clinical decision-making.

## Data availability statement

Publicly available datasets were analyzed in this study. This data can be found here: https://portal.gdc.cancer.gov, TCGA-PRAD https://www.ncbi.nlm.nih.gov/gds,GSE46602 and GSE70770.

## Ethics statement

The studies involving human participants were reviewed and approved by the Ethics Committee of the Institutional Review Board of the Second Affiliated Hospital of Nanchang University. The patients/participants provided their written informed consent to participate in this study.

## Author contributions

ZY designed this study. Data acquisition and analysis: ZY, HC and FX. Experiments: ZY and FX. Original draft: ZY. Review and editing: ZY, LD, ZS, TZ. Supervision and administration: TZ. All authors contributed to the article and approved the submitted version.

## References

[1] Xia C Dong X Li H Cao M Sun D He S. Cancer statistics in China and united states, 2022: Profiles, trends, and determinants. Chin Med J (2022) 135(5):584–90. doi: 10.1097/cm9.0000000000002108 PMC8920425 35143424

[2] Siegel RL Miller KD Fuchs HE Jemal A. Cancer statistics, 2022. CA: Cancer J Clin (2022) 72(1):7–33. doi: 10.3322/caac.21708 35020204

[3] Ilic D Evans SM Allan CA Jung JH Murphy D Frydenberg M. Laparoscopic and robotic-assisted versus open radical prostatectomy for the treatment of localised prostate cancer. Cochrane Database systematic Rev (2017) 9(9):Cd009625. doi: 10.1002/14651858.CD009625.pub2 PMC6486168 28895658

[4] Tourinho-Barbosa R Srougi V Nunes-Silva I Baghdadi M Rembeyo G Eiffel SS. Biochemical recurrence after radical prostatectomy: What does it mean? Int Braz J urol (2018) 44(1):14–21. doi: 10.1590/s1677-5538.Ibju.2016.0656 29039897 PMC5815528

[5] Van den Broeck T van den Bergh RCN Arfi N Gross T Moris L Briers E. Prognostic value of biochemical recurrence following treatment with curative intent for prostate cancer: A systematic review. Eur Urol (2019) 75(6):967–87. doi: 10.1016/j.eururo.2018.10.011 30342843

[6] Marra G Karnes RJ Calleris G Oderda M Alessio P Palazzetti A. Oncological outcomes of salvage radical prostatectomy for recurrent prostate cancer in the contemporary era: A multicenter retrospective study. Urologic Oncol (2021) 39(5):296.e21–.e29. doi: 10.1016/j.urolonc.2020.11.002 33436329

[7] Ogaya-Pinies G Linares-Espinos E Hernandez-Cardona E Jenson C Cathelineau X Sanchez-Salas R. Salvage robotic-assisted radical prostatectomy: Oncologic and functional outcomes from two high-volume institutions. World J Urol (2019) 37(8):1499–505. doi: 10.1007/s00345-018-2406-4 30006908

[8] Tilki D Preisser F Graefen M Huland H Pompe RS. External validation of the European association of urology biochemical recurrence risk groups to predict metastasis and mortality after radical prostatectomy in a European cohort. Eur Urol (2019) 75(6):896–900. doi: 10.1016/j.eururo.2019.03.016 30955970

[9] Pei XM Yeung MHY Wong ANN Tsang HF Yu ACS Yim AKY. Targeted sequencing approach and its clinical applications for the molecular diagnosis of human diseases. Cells (2023) 12(3):493. doi: 10.3390/cells12030493 36766834 PMC9913990

[10] Cui M Cheng C Zhang L. High-throughput proteomics: A methodological mini-review. Lab Invest (2022) 102(11):1170–81. doi: 10.1038/s41374-022-00830-7 PMC9362039 35922478

[11] Tang FH Xue C Law MY Wong CY Cho TH Lai CK. Prognostic prediction of cancer based on radiomics features of diagnostic imaging: The performance of machine learning strategies. J digital Imaging (2023). doi: 10.1007/s10278-022-00770-0 PMC10287586 36781589

[12] Yan C Gong B Wei Y Gao Y. Deep multi-view enhancement hashing for image retrieval. IEEE Trans Pattern Anal Mach Intell (2021) 43(4):1445–51. doi: 10.1109/tpami.2020.2975798 32091992

[13] Li H Cheng ZJ Liang Z Liu M Liu L Song Z. Novel nutritional indicator as predictors among subtypes of lung cancer in diagnosis. Front Nutr (2023) 10:1042047. doi: 10.3389/fnut.2023.1042047 36776604 PMC9909296

[14] Yan C Hao Y Li L Yin J Liu A Mao Z. Task-adaptive attention for image captioning. IEEE Trans IEEE Trans Circuits Syst Video Technol (2021) 32(1):43–51. doi: 10.1109/TCSVT.2021.3067449

[15] Elias MH Das S Abdul Hamid N. Candidate genes and pathways in cervical cancer: A systematic review and integrated bioinformatic analysis. Cancers (Basel) (2023) 15(3):853. doi: 10.3390/cancers15030853 36765810 PMC9913780

[16] Chen L Wang C Sun H Wang J Liang Y Wang Y. The bioinformatics toolbox for circrna discovery and analysis. Briefings Bioinf (2021) 22(2):1706–28. doi: 10.1093/bib/bbaa001 PMC7986655 32103237

[17] Yin X Wang P Yang T Li G Teng X Huang W. Identification of key modules and genes associated with breast cancer prognosis using wgcna and cerna network analysis. Aging (2020) 13(2):2519–38. doi: 10.18632/aging.202285 PMC7880379 33318294

[18] Rezaei Z Ranjbaran J Safarpour H Nomiri S Salmani F Chamani E. Identification of early diagnostic biomarkers *Via* wgcna in gastric cancer. Biomed pharmacother = Biomed pharmacother (2022) 145:112477. doi: 10.1016/j.biopha.2021.112477 34864309

[19] Liesenfeld L Kron M Gschwend JE Herkommer K. Prognostic factors for biochemical recurrence more than 10 years after radical prostatectomy. J Urol (2017) 197(1):143–8. doi: 10.1016/j.juro.2016.07.004 27418452

[20] Aurilio G Cimadamore A Mazzucchelli R Lopez-Beltran A Verri E Scarpelli M. Androgen receptor signaling pathway in prostate cancer: From genetics to clinical applications. Cells (2020) 9(12):2653. doi: 10.3390/cells9122653 33321757 PMC7763510

[21] Koike H Sekine Y Kamiya M Nakazato H Suzuki K. Gene expression of survivin and its spliced isoforms associated with proliferation and aggressive phenotypes of prostate cancer. Urology (2008) 72(6):1229–33. doi: 10.1016/j.urology.2007.12.064 18336887

[22] Wheatley SP Altieri DC. Survivin at a glance. J Cell Sci (2019) 132(7):jcs223826. doi: 10.1242/jcs.223826 30948431 PMC6467487

[23] Ma T Gu J Wen H Xu F Ge D. Birc5 modulates pd-L1 expression and immune infiltration in lung adenocarcinoma. J Cancer (2022) 13(10):3140–50. doi: 10.7150/jca.69236 PMC9414029 36046648

[24] Conde M Michen S Wiedemuth R Klink B Schröck E Schackert G. Chromosomal instability induced by increased Birc5/Survivin levels affects tumorigenicity of glioma cells. BMC Cancer (2017) 17(1):889. doi: 10.1186/s12885-017-3932-y 29282022 PMC5745881

[25] Martínez-Sifuentes MA Bassol-Mayagoitia S Nava-Hernández MP Ruiz-Flores P Ramos-Treviño J Haro-Santa Cruz J. Survivin in breast cancer: A review. Genet testing Mol Biomarkers (2022) 26(9):411–21. doi: 10.1089/gtmb.2021.0286 36166738

[26] Philip M Schietinger A. Cd8(+) T cell differentiation and dysfunction in cancer. Nat Rev Immunol (2022) 22(4):209–23. doi: 10.1038/s41577-021-00574-3 PMC9792152 34253904

[27] Qiao J Liu Z Dong C Luan Y Zhang A Moore C. Targeting tumors with il-10 prevents dendritic cell-mediated Cd8(+) T cell apoptosis. Cancer Cell (2019) 35(6):901–15.e4. doi: 10.1016/j.ccell.2019.05.005 31185213

[28] Lynn GM Sedlik C Baharom F Zhu Y Ramirez-Valdez RA Coble VL. Peptide-Tlr-7/8a conjugate vaccines chemically programmed for nanoparticle self-assembly enhance Cd8 T-cell immunity to tumor antigens. Nat Biotechnol (2020) 38(3):320–32. doi: 10.1038/s41587-019-0390-x PMC7065950 31932728

[29] Yanai Y Kosaka T Mikami S Hongo H Yasumizu Y Takeda T. Cd8-positive T cells and Cd204-positive M2-like macrophages predict postoperative prognosis of very high-risk prostate cancer. Sci Rep (2021) 11(1):22495. doi: 10.1038/s41598-021-01900-4 34795362 PMC8602636

[30] Liang J Zhao W Tong P Li P Zhao Y Li H. Comprehensive molecular characterization of inhibitors of apoptosis proteins (Iaps) for therapeutic targeting in cancer. BMC Med Genomics (2020) 13(1):7. doi: 10.1186/s12920-020-0661-x 31964418 PMC6975060

[31] Zhao Y Liu S Li S Zhang G Tian A Wan Y. Birc5 regulates inflammatory tumor microenvironment-induced aggravation of penile cancer development *in vitro* and *in vivo* . BMC Cancer (2022) 22(1):448. doi: 10.1186/s12885-022-09500-9 35461228 PMC9035256

