# Supplementary figures and images for: Identification of a prognostic biomarker predicting biochemical recurrence and construction of a novel nomogram for prostate cancer
Source: Front Oncol. 2023 Apr 3;13:1115718. doi: 10.3389/fonc.2023.1115718 (PMC10106702; doi:10.3389/fonc.2023.1115718)

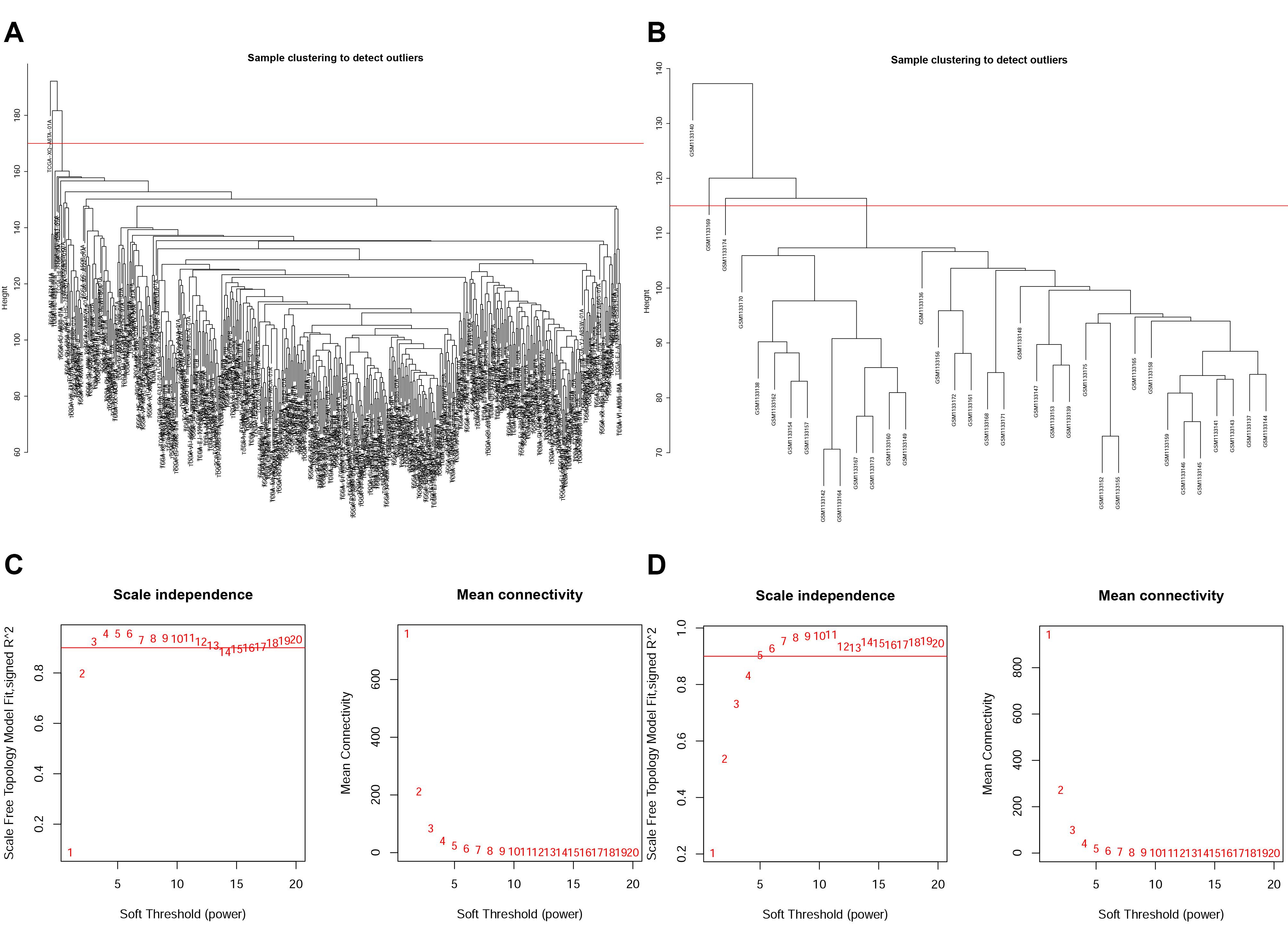

Supplement: Supplementary file 1 [file Image_1.tif]

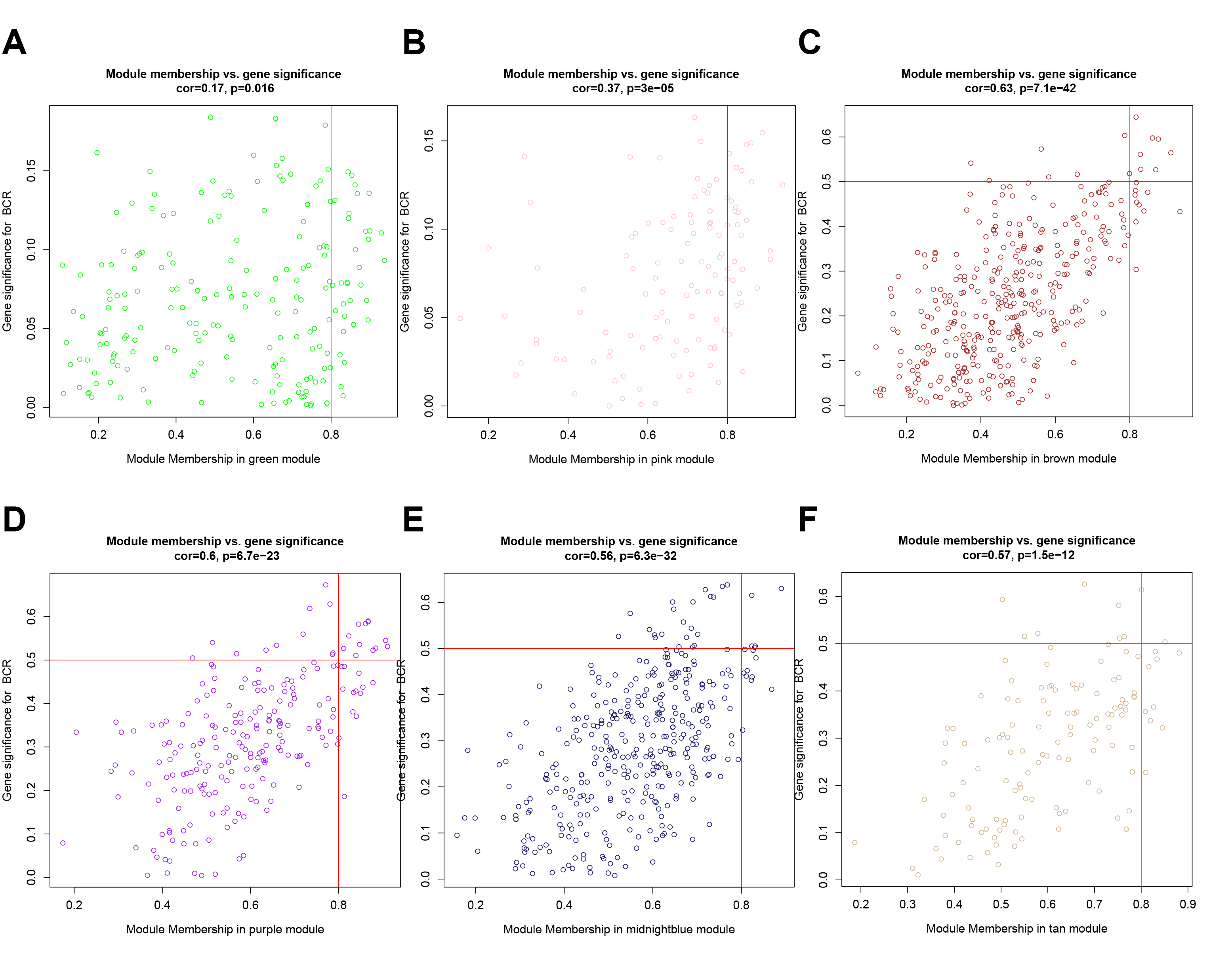

Supplement: Supplementary file 2 [file Image_2.tif]

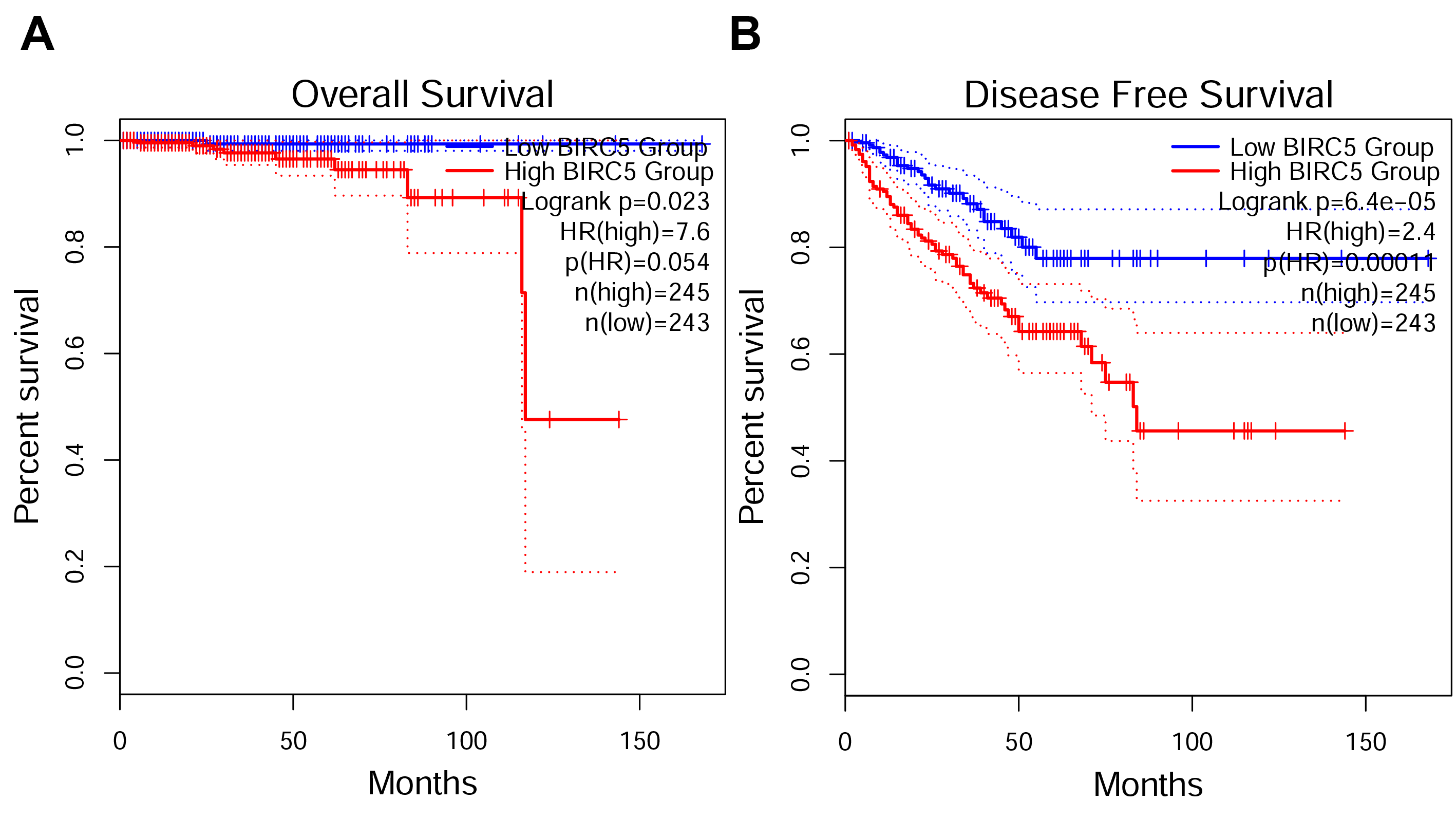

Supplement: Supplementary file 3 [file Image_3.tif]

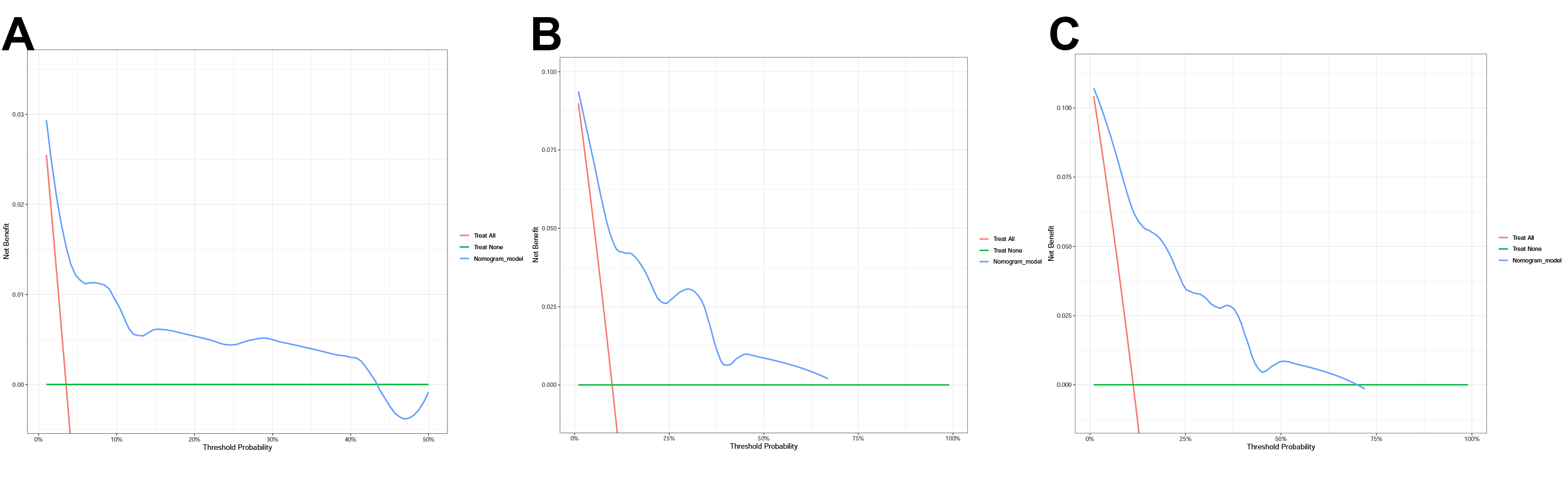

Supplement: Supplementary file 4 [file Image_4.tif]
