# Supplementary material for: Identification of a prognostic biomarker predicting biochemical recurrence and construction of a novel nomogram for prostate cancer
Source: Front Oncol. 2023 Apr 3;13:1115718. doi: 10.3389/fonc.2023.1115718 (PMC10106702; doi:10.3389/fonc.2023.1115718)
Supplement: Supplementary file 5 [file Table_1.docx]

**Table S1:** The primer sequences of BIRC5 and ACTIN

| **Primer** | **Forward Sequence** | **Reverse Sequence** |
| --- | --- | --- |
| BIRC5 | CAG TGG CTG CTT CTC TCT C | ACT GCC TTC TTC CTC CCT |
| ACTIN | TCTCCCAAGTCCACACAGG | GGCACGAAGGCTCATCA |
