# Supplementary material for: Identification of a prognostic biomarker predicting biochemical recurrence and construction of a novel nomogram for prostate cancer
Source: Front Oncol. 2023 Apr 3;13:1115718. doi: 10.3389/fonc.2023.1115718 (PMC10106702; doi:10.3389/fonc.2023.1115718)
Supplement: Supplementary file 9 [file Table_5.docx]

**Table S5:** BCR status of patients selected in all datasets.

| Dataset | Number of BCR patients | Number of BCR-Free patients | BCR-Time (Median-months) | Total |
| --- | --- | --- | --- | --- |
| TCGA-PRAD | 27 | 369 | 29.417 | 396 |
| GSE46602 | 14 | 22 | 31.885 | 36 |
| GSE70770 | 62 | 137 | 36.690 | 199 |
