# Supplementary material for: Identification of a prognostic biomarker predicting biochemical recurrence and construction of a novel nomogram for prostate cancer
Source: Front Oncol. 2023 Apr 3;13:1115718. doi: 10.3389/fonc.2023.1115718 (PMC10106702; doi:10.3389/fonc.2023.1115718)
Supplement: Supplementary file 10 [file Table_6.docx]

Legends:

Supplementary Figure S1: WGCNA analysis. Hierarchical clustering analysis for excluding outlier samples in TCGA-PRAD dataset (A) and GSE46602 dataset (B). The scale-free fit index and the mean connectivity for various soft-thresholding powers in the TCGA-PRAD dataset (C) and GSE46602 dataset (D).

Supplementary Figure S2: The correlation modules in the TCGA-PRAD dataset and GSE46602 dataset. (A) The gene correlation scatter plots of the green module in the TCGA-PRAD dataset. (B) The gene correlation scatter plots of the pink module in the TCGA-PRAD dataset. (C) The gene correlation scatter plots of the brown module in the GSE46602 dataset. (D) The gene correlation scatter plots of the purple module in the GSE46602 dataset. (E) The gene correlation scatter plots of the midnight-blue module in the GSE46602 dataset. (F) The gene correlation scatter plots of the tan module in the GSE46602 dataset.

Supplementary Figure S3: K-M survival analysis in GEPIA database. (A) K-M overall survival (OS) analysis. (B) K-M disease-free survival (DFS) analysis.

Supplementary Figure S4: Decision curve analysis (DCA) for nomogram model. (A) 1-year DCA curve. (B) 3-year DCA curve. (C) 5-year DCA curve.
